# Supplementary material for: Preventive Measures for SARS-CoV-2 in the Workplace and Vaccine Acceptance: Assessment of Knowledge, Attitudes and Behaviors of Workers in Southern Italy
Source: Vaccines (Basel). 2022 Nov 5;10(11):1872. doi: 10.3390/vaccines10111872 (PMC9694280; doi:10.3390/vaccines10111872)
Supplement: Supplementary file 1 [file vaccines-10-01872-s001.zip › vaccines-1985705-supplementary.pdf]

Variables included in the logistic regression models with related categories

Model 1. Knowledge of SARS-CoV-2 routes of transmission and preventive measures

Independent variables	Code
Age	Years (continuos)
Gender	Male = 0<br>Female = 1
Sons/daughters	None = 0<br>≥1 = 1
Number of cohabitants	None = 0<br>≥1 = 1
Education level	Other = 0<br>University degree = 1
Employment type	Other = 0<br>Manager/Office employee = 1
Working from home during pandemic	No = 0<br>Yes = 1
Years of working activity	Years (continuos)
Having been infected with SARS-CoV-2	No = 0<br>Yes = 1
Having had cohabitants infected with SARS-CoV-2	No = 0<br>Yes = 1
Self-rated health status	Low (1-7) = 0<br>High (8-10) = 1
Having received information about COVID-19 prevention in the workplace from companies	No = 0<br>Yes = 1
Need for further information on COVID-19 prevention or COVID-19 vaccine	No = 0<br>Yes = 1

Model 2. High concern of contracting and transmitting SARS-CoV-2 to family

Independent variables	Code
Age	Years (continuos)
Gender	Male = 0<br>Female = 1
Sons/daughters	None = 0<br>≥1 = 1
Number of cohabitants	None = 0<br>≥1 = 1
Education level	Other = 0<br>University degree = 1
Employment type	Other = 0<br>Manager/Office employee = 1
Working from home during pandemic	No = 0<br>Yes = 1
Years of working activity	Years (continuos)
Having had cohabitants infected with SARS-CoV-2	No = 0<br>Yes = 1
Self-rated health status	Low (1-7) = 0<br>High (8-10) = 1
Having received information about COVID-19 prevention in the workplace from companies	No = 0<br>Yes = 1
Need for further information on COVID-19 prevention or COVID-19 vaccine	No = 0<br>Yes = 1
Knowledge of all SARS-CoV-2 modes of transmission and main preventive measures	No = 0<br>Yes = 1
Knowing main COVID-19 symptoms	Others = 0<br>Fever/Cough/Tiredness = 1

Model 3. Adherence to all preventive measures in the workplace

Independent variables	Code
Age	Years (continuos)
Gender	Male = 0<br>Female = 1
Sons/daughters	None = 0<br>≥1 = 1
Number of cohabitants	None = 0<br>≥1 = 1
Education level	Other = 0<br>University degree = 1
Employment type	Other = 0<br>Manager/Office employee = 1
Working from home during pandemic	No = 0<br>Yes = 1
Years of working activity	Years (continuos)
Having had cohabitants infected with SARS-CoV-2	No = 0<br>Yes = 1
Self-rated health status	Low (1-7) = 0<br>High (8-10) = 1
Having received information about COVID-19 prevention in the workplace from companies	No = 0<br>Yes = 1
Need for further information on COVID-19 prevention or COVID-19 vaccine	No = 0<br>Yes = 1
Knowledge of all SARS-CoV-2 routes of transmission and main preventive measures	No = 0<br>Yes = 1
Knowing main COVID-19 symptoms	Others = 0<br>Fever/Cough/Tiredness = 1
Belief that the COVID-19 vaccine is highly useful and safe	No = 0<br>Yes = 1
Belief that use of masks in the workplace reduces risk of SARS-CoV-2 transmission	Uncertain/Disagree = 0<br>Agree = 1
Belief that physical distancing in the workplace reduces risk of SARS-CoV-2 transmission	Uncertain/Disagree = 0<br>Agree = 1
Belief that the COVID-19 vaccine also protects family from contagion	Uncertain/Disagree = 0<br>Agree = 1
Belief that the vaccine offers high protection against COVID-19	Uncertain/Disagree = 0<br>Agree = 1
Being highly concerned of contracting and transmitting SARS-CoV-2 to family	No = 0<br>Yes = 1

Model 4. Unwillingness to get vaccinated for COVID-19 because the vaccination was considered not useful and/or safe and/or effective

Independent variables	Code
Age	Years (continuos)
Gender	Male = 0<br>Female = 1
Sons/daughters	None = 0<br>≥1 = 1
Number of cohabitants	None = 0<br>≥1 = 1
Education level	Other = 0<br>University degree = 1
Employment type	Other = 0<br>Manager/Office employee = 1
Working from home during pandemic	No = 0<br>Yes = 1
Years of working activity	Years (continuos)

Having had cohabitants infected with SARS-CoV-2	No = 0<br>Yes = 1
Self-rated health status	Low (1-7) = 0<br>High (8-10) = 1
Having received information about COVID-19 prevention in the workplace from companies	No = 0<br>Yes = 1
Need for further information on COVID-19 prevention or COVID-19 vaccine	No = 0<br>Yes = 1
Knowledge of all SARS-CoV-2 routes of transmission and main preventive measures	No = 0<br>Yes = 1
Knowing main COVID-19 symptoms	Others = 0<br>Fever/Cough/Tiredness = 1
Belief that the COVID-19 vaccine is highly useful and safe	Low (1-7) = 0<br>High (8-10) = 1
Belief that use of masks in the workplace reduces risk of SARS-CoV-2 transmission	Uncertain/Disagree = 0<br>Agree = 1
Belief that physical distancing in the workplace reduces risk of SARS-CoV-2 transmission	Uncertain/Disagree = 0<br>Agree = 1
Belief that the COVID-19 vaccine also protects family from contagion	Uncertain/Disagree = 0<br>Agree = 1
Belief that the vaccine offers high protection against COVID-19	Uncertain/Disagree = 0<br>Agree = 1
Being highly concerned of contracting and transmitting SARS-CoV-2 to family	No = 0<br>Yes = 1
